# Key factors for promoting protective behaviours in future pandemics: an umbrella review

**DOI:** 10.1186/s13690-026-01948-6

**Published:** 2026-06-17

**Authors:** Aurélien Cornil, Sandrine Roussel, Marie Vander Haegen, Catherine Grenier, Dominique Vanpee, Stephan Van den Broucke

**Affiliations:** 1 Adaptation, Resilience and Change (ARCh), Faculty of Psychology, Speech and Language Therapy and Education (FPLSE), ULiège, Liège, 4000 Belgium; 2 Institute of Health and Society (IRSS), Woluwe-Saint-Lambert, UCLouvain, 1200 Belgium; 3 Laboratoire de Psychologie des Pays de la Loire (LPPL), Nantes Université, Nantes, 44312 France; 4 Psychological Sciences Research Institute (IPSY), UCLouvain, Louvain-la-Neuve, 1348 Belgium; 5 Research Unit for a Life-Course perspective on Health & Education (RUCHE), ULiège, Liège, 4000 Belgium; 6 Laboratoire de Psychopathologie et Processus de Santé (LPPS), Université de Paris Cité, Paris, 75006 France; 7 Centre Hospitalier Universitaire UCLouvain Namur, Yvoir, 5530 Belgium

**Keywords:** COVID-19, Disease outbreaks, Pandemic, Dpidemic, Public health, Protective behaviours, Enabling factors, Barriers, Review

## Abstract

**Background:**

The COVID-19 pandemic highlighted the critical importance of protective behaviours—such as mask-wearing, hand hygiene, and physical distancing—in managing public health crises. Understanding the factors that influence these behaviours is essential for improving preparedness and response in future health crises. This umbrella review aimed to synthesize evidence on determinants of protective behaviours during pandemics and to provide recommendations for policy and practice.

**Methods:**

Following PRISMA 2020 guidelines, we conducted an umbrella review of peer-reviewed literature reviews and meta-analyses published up to June 2025. Searches in Epistemonikos, MEDLINE, and Scopus targeted reviews on “protective behaviours” and “COVID-19”, including other infectious diseases. Eligibility criteria followed the Population–Concept–Context framework: general population, multifactorial determinants of protective behaviours, and pandemic contexts. Data extraction was performed in a table that included review characteristics, behavioural domains (general compliance, medical interventions, open dialogue promotion, information handling, hygiene, physical distancing, and other behaviours), and factor valence. We conducted a narrative synthesis organizing factors by sociodemographic, personal, and social-environmental categories, with valence classification based on consistency of findings across reviews. Quality appraisal used the Joanna Briggs Institute checklist.

**Results:**

From 86 records identified, 11 met inclusion criteria, covering COVID-19 and other diseases (e.g., H1N1, SARS, MERS, Ebola). Quality scores averaged 8.8/10. Protective behaviours were influenced by three categories of factors: (1) sociodemographic (age, gender, education, socio-economic status) (2), personal (perceptions, beliefs, trust in authorities and science, political orientation) and (3) social/environmental (access to protective materials, social norms, community support, health education and communication, policies). Enabling factors included trust in credible sources, perceived effectiveness of measures, and multimodal communication. Barriers comprised misinformation, conspiracy beliefs, and resource inaccessibility. Some factors (e.g., age, gender, education) showed inconsistent effects across behaviours. Findings underscore the need for culturally sensitive messaging, transparent communication, and targeted interventions for vulnerable populations.

**Conclusions:**

This umbrella review identifies multi-level determinants of protective behaviours to inform pandemic preparedness. It highlights the need for transparent communication through trusted channels, culturally adapted messaging, equitable access to protective resources, and targeted interventions for low-engagement groups. Integrating these determinants early may enhance population-level adherence.

**Registration:**

PROSPERO registration number: CRD42023467236.

**Supplementary Information:**

The online version contains supplementary material available at https://doi.org/10.1186/s13690-026-01948-6.

**Table:** 

Text box 1. Contributions to the literature
• Synthesizes evidence from 11 reviews across seven behavioural domains, offering a comprehensive overview of factors influencing protective behaviours during pandemics.
• Identifies multi-level determinants (sociodemographic, psychological, and social/environmental) and categorizes them as enabling, hindering, or neutral, supporting targeted interventions.
• Highlights inconsistencies and potential effect modifiers, providing insights into heterogeneity and guiding context-sensitive policy design.
• Offers actionable recommendations for policymakers, emphasizing trust-building, culturally appropriate communication, and equitable resource distribution.
• Addresses gaps in previous literature by integrating findings from diverse pandemics and proposing directions for future research on behavioural determinants.

## Introduction

The COVID-19 pandemic of 2020 highlighted the vulnerabilities of a globally interconnected society to various crises [1]. The pandemic underscored the limitations of biomedical interventions, and the need for social and behavioural strategies alongside them. As was also the case for previous outbreaks, measures to curb the pandemic such as physical distancing, mask-wearing, and vaccination necessitated behavioural adjustments from both citizens and health professionals, and called for community cooperation and supportive collective action [2–4], especially as scepticism regarding the health system’s efficacy and perceptions of unjust or unnecessary measures intensified individuals’ reactance against protective behaviours. Therefore, effective management of a health crisis requires a careful integration of communication, information dissemination, and individual support.

Experience with previous epidemics illustrates the dual necessity of health and social management in epidemic control [5]. This involves six strategic components: virus transmission control, maintaining healthcare quality, preventive measures in various environments, managing imported cases, risk reduction in vulnerable settings, and promoting community responsibility through sanitary practices. Within this broader framework, promoting protective behaviours represents a critical yet complex challenge, as it requires understanding and influencing individual and collective actions across diverse populations and contexts. Unlike surveillance or vaccine development, which are primarily technical and institutional functions, protective behaviours represent the primary means by which individuals can directly contribute to outbreak control, particularly in the early phases before medical interventions are widely available. They depend on public engagement and adherence, making them particularly sensitive to social, psychological, and environmental factors. This review therefore focuses specifically on understanding what enables or hinders protective behaviours, as this knowledge is essential for effective implementation of the broader pandemic response strategy.

The significance of protective behaviours has been well-established in scientific research, not least during COVID-19. To promote individual protective behaviours, authorities typically use a mix of information and awareness campaigns, and legislative approaches. Analysing the psychological and social factors that influence these behaviours can enhance intervention effectiveness [5–9]. Protective behaviours are shaped by cognitive, emotional, and social influences. Public health messaging must clearly convey disease risks and preventive benefits. Although studies on protective behaviour adoption during epidemics yield inconsistent results due to different methodologies used, several reviews have consolidated insights on the factors that affect the adoption of protective behaviour [10–12]. Of these, a high perceived risk of infection combined with trust in information sources appears to be of crucial influence [8, 9, 13].

Despite the proliferation of reviews on protective behaviours during COVID-19, several gaps justify an umbrella review synthesis. First, existing reviews have predominantly focused on single behaviours (e.g., mask-wearing only) or specific populations (e.g., healthcare workers), limiting cross-behaviour and cross-population insights. Second, most reviews examined COVID-19 in isolation, missing opportunities to identify patterns across multiple pandemic contexts (H1N1, SARS, MERS, Ebola). Third, the sheer volume of reviews (> 60,000 COVID-19 reviews published between January 2020 and June 2025; source: MEDLINE) makes it challenging for policymakers and practitioners to identify consistent, actionable determinants. An umbrella review addresses these gaps by: (1) synthesizing evidence across multiple behaviours and contexts (2), identifying converging and diverging findings across reviews (3), assessing the consistency and quality of evidence, and (4) providing consolidated, evidence-based guidance for future pandemic preparedness. This higher-order synthesis is essential for translating the extensive but fragmented review literature into coherent policy recommendations.This paper aims to synthesize findings on actionable policy recommendations during pandemics by presenting an umbrella review compiling the results from various studies into an accessible format [14, 15]. By integrating evidence on facilitators and barriers to different protective behaviours, it can enhance the understanding of the subject and guide government interventions during future public health crises. The purpose of this umbrella review is to answer the following question: “What factors enabled or hindered protective behaviours during pandemics and infectious disease outbreaks, with a primary focus on COVID-19 but also including evidence from previous pandemics?”

## Methods

### Protocol and registration

This umbrella review followed the PRISMA 2020 checklists, available in Supplementary File 1. The research protocol is registered on PROSPERO, the international prospective register of systematic reviews of the National Institute for Health Research (registration number: CRD42023467236; https://www.crd.york.ac.uk/PROSPERO/view/CRD42023467236).

### Search strategy

An umbrella review following the Preferred Reporting Items for Systematic Reviews and Meta-Analyses (PRISMA) 2020 guidelines [16] was conducted on published literature reviews and meta-analyses regarding protective behaviours and COVID-19. As recommended by Goossen and colleagues [17], the Epistemonikos, MEDLINE databases were consulted, along with Scopus as additional database. Grey literature (unpublished reviews, organizational reports, dissertations, preprints) was excluded because of potential lack of rigor and transparence in the methodology, and because of resource constraints. We used “COVID-19” and “protective behaviours” as search terms, accommodating for variations in American and British English. Filters were applied to identify (systematic) reviews and meta-analyses (for queries, see Supplementary File 2). No time limit was set for the research, but it was expected that there would be no results before 2020 due to the emergence of COVID-19. The search occurred in November 2023 and was updated in June 2025.

We acknowledge that our search strategy was deliberately focused rather than exhaustive. Our search strategy was designed to identify reviews that explicitly synthesized evidence on protective behaviours during COVID-19 and infectious diseases outbreaks. Umbrella reviews synthesize existing systematic reviews rather than primary studies and therefore require methodological approaches tailored to that focus [18, 19] requiring distinct procedures for search, selection, and synthesis compared to primary-study systematic reviews. Moreover, the COVID-19 pandemic generated an unprecedented volume of reviews, and focused research can be a reasonable trade-off given the resources available, provided that it is transparent [20, 21].

The term “protective behaviours” is used to describe actions such as wearing masks, maintaining physical distance, or practicing hand hygiene, and other actions aimed at minimizing the risk of infection [22, 23]. Other search terms (e.g., health behaviours, adherence, vaccination) were tested but yielded unmanageable results, with many irrelevant reports (e.g., influence of COVID-19 on health behaviours), resulting in our final choice.

While our focus was COVID-19, we wanted to include other infectious diseases that were discussed to bring more insights regarding protective factors against COVID-19, which is why we only used “COVID-19” as a context but deemed the data about other diseases eligible.

### Framework and eligibility criteria

The “Population-Concept-Context” framework [24] and eligibility criteria are presented in Table 1.

**Table 1:** Population-concept-context framework and study designs for eligibility criteria

	Inclusion Criteria	Exclusion Criteria
Population	General population globally or sub-populations (humans from all age groups)	Specific clinical populations (e.g., hospitalized patients, people suffering from psychopathology, immunocompromised individuals)
Concept	Multifactorial determinants of protective behaviours adoption during infectious disease outbreaks • Scope: Facilitators, barriers, and ineffective factors of protective behaviours against Covid-19 or other infectious diseases considered in reviews related to COVID-19 • Types: All factor categories (e.g., psychological, social, environmental, economic, cultural, interventions) • Behaviours: Protective behaviours (including behaviours perceived as protective, without scientific consensus, such as hoarding) and adherence to recommendations, and specific measures (e.g., mask wearing, physical distancing, hand hygiene)	No link between factors and protective behaviours Intentions or attitudes towards protective behaviours Retroactive effects of protective behaviours (e.g., the influence of mask wearing on distancing)
Context	Covid-19 pandemic and other infectious diseases • Temporal: Throughout pandemic phases (no distinction between early/later stages) • Geographic: Global scope	Non-infectious disease contexts
Study Design	Peer reviewed review studies or meta-analyses • Language: English, French, Dutch	Grey literature, conference outputs, books, preprints, primary studies, opinion pieces, theses (i.e., not peer reviewed)

### Data synthesis

The selection and screening of the reviews followed the PRISMA 2020 guidelines [16]. Titles and abstracts of all search results were independently screened by two researchers (AC and SR) against eligibility criteria and organized into three categories: yes, no, maybe. Disagreements were resolved through discussion, and with a third reviewer (CG) when consensus could not be reached. Full-text articles of potentially eligible studies (*n* = 21) were retrieved and checked by one of the two initial reviewers (AC or SR). Exclusion of reports were systematically discussed to verify the reason of rejection; there was no disagreement for this stage. We then conducted a narrative synthesis in four stages.

First, extracted data from included reviews were organized into a comprehensive matrix (Supplementary File 3) by one reviewer (AC or SR). Individual reviews were set in rows (*n* = 11) and columns presented the reference, the type of pandemic/epidemic, the population/sample, the method, the theoretical framework, the objective of the review, the determinant factors (enabling, blocking, and with no relation, i.e., with no statistically significant corelation), and review quality and risk of bias assessment identified in each review. This matrix allowed systematic comparison of findings across reviews.

Second, quality assessment was performed by one researcher (AC) and SciSpace [25] separately with Joanna Briggs Institute (JBI) critical appraisal checklist for systematic reviews and research syntheses [18]. SciSpace was asked to evaluate the 11 papers based on JBI criteria and to justify its assessment with contextualized citations. The researcher (AC) then confronted both analyses and decided on the final appraisal in a human-led process (Supplementary File 4).

Third, another table reorganized and integrated the factors from the first matrix according to each category of protective behaviours: (1) general protective behaviours/adherence/compliance with public health recommendations, (2) medical interventions, (3) open dialogue promotion, (4) information, (4) hygiene and transmission prevention, (5) distancing, and (6) other behaviours (i.e., non-evidence-based strategies adopted by portions of the population such as hoarding). The latter were included for descriptive completeness and to understand the full spectrum of behavioural responses but are not considered relevant to the discussion. These categories, when relevant, included more specific behaviours (e.g., vaccination, mask, isolation). As in the first matrix, the factors were classified as enabling, hindering, and factors with no relation. Eventually, ambiguous factors (i.e., reported in at least two classifications) were set apart and highlighted for further analyses.

Fourth, through iterative discussion among the research team (AC, SR, SVdB), we conducted a narrative synthesis of the determinant factors inductively grouped into three overarching categories: (1) sociodemographic factors, (2) personal factors, and (3) social and environmental factors. Controversial results (i.e., factors reported in at least two valences) were interpreted by using the number of studies (e.g., more studies in favour of “enabling factor”), the context (type of pandemic, general or specific population), and quality score.

This synthesis approach prioritized transparency and acknowledged uncertainty where it existed, consistent with umbrella review methodology.

### Consideration of effect modifiers and heterogeneity

Because this umbrella review synthesizes findings from systematic reviews rather than primary studies, no quantitative heterogeneity analysis was conducted. Instead, potential sources of variability were examined qualitatively. The included reviews differed in their populations (e.g., general population, healthcare workers, displaced communities), pandemic contexts (e.g., COVID-19, H1N1, SARS, Ebola), cultural settings, and methodological approaches (e.g., scoping, systematic, rapid reviews). These contextual differences, along with variations in how protective behaviours and determinants were defined and measured, were considered when interpreting the consistency of reported associations. Effect modifiers such as sociodemographic factors, psychological characteristics, and resource availability were therefore examined as contextual influences rather than as variables for stratified analysis. This qualitative consideration allowed us to acknowledge heterogeneity across reviews while avoiding over-interpretation of divergent findings.

## Results

### Overview of the search results

**Fig. 1:**
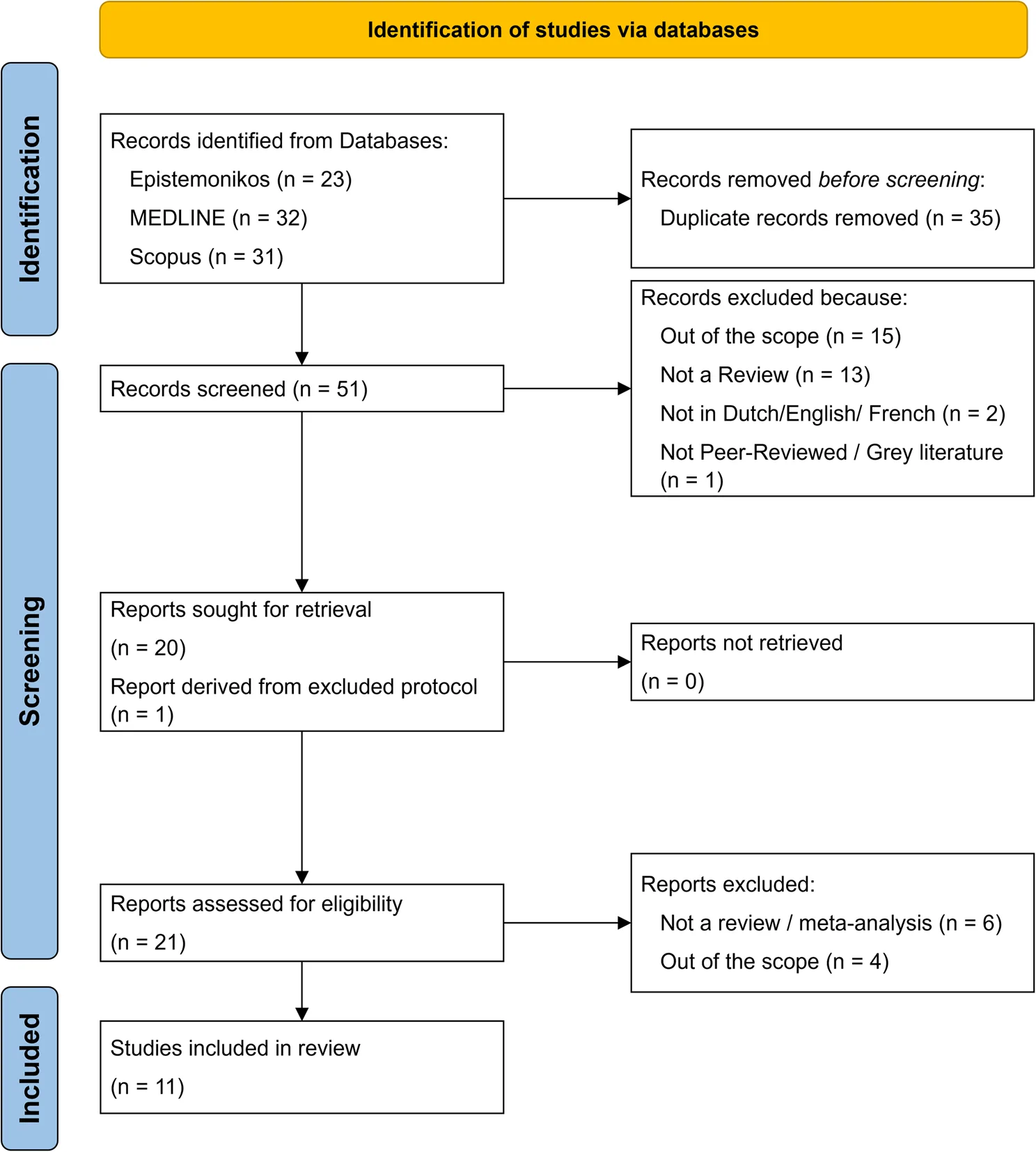
PRISM 2020 flow diagram of study selection for this umbrella review

The search yielded 23 records from Epistemonikos, 32 from Medline, and 31 from Scopus (Fig. 1). Following the removal of 35 duplicates, 51 records were screened based on titles and abstracts. Of these, 31 articles were excluded because they were out of the scope (*n* = 15), not a review (*n* = 13), not in selected languages (*n* = 2), and not peer-reviewed / grey literature (*n* = 1). The 20 reports sought for retrieval were assessed for eligibility. One protocol was identified and excluded, but the corresponding completed report was included as a full record. The evaluation of the full texts led to the exclusion of 10 additional reports, with 6 not qualifying as reviews or meta-analyses (i.e., no proper methodology) and 4 being out of this review’s scope. Consequently, 11 reviews were deemed suitable for inclusion and data extraction.

### Description of selected reviews

The data from the selected reviews and the quality assessment are respectively detailed in Supplementary File 3 and 4. The eleven reviews, published from 2020 to 2025, encompass various types: rapid scoping [26], systematic [27–30], integrative [31], rapid [32, 33], rapid evidence review [34], scoping [35], and integrative rapid narrative [36]. The number of articles in these studies ranges from 13 to 78, with a mean of 44.09. In addition to COVID-19, other diseases examined include H1N1 [26, 28, 30, 33, 34, 36], influenza [26, 30, 31, 33, 34], SARS [28, 34, 36], MERS [28, 33, 36], Ebola [28], HIV, hepatitis, Zika [33], tuberculosis, pertussis [30], other respiratory viruses [34], and hypothetical pandemics [36]. One study [31] specifically addresses seasonal influenza among healthcare workers to offer recommendations for COVID-19.

The methodologies of the studies are articulated in all reviews, with ten of them specifying the countries from which data were collected, spread across the following continents: Africa [26, 28, 30], Asia [26–30, 34–36], Europe [26, 28–30, 32, 36], North America [26, 28–30, 32, 34, 36], Oceania [26, 28–30, 32, 34, 36], and South America [29, 30]. Four reviews incorporate theoretical frameworks, including health behaviour change models [31], health belief model [30], behaviour change wheel [30, 35, 36], the Capability-Opportunity-Motivation-Behaviour framework [35] and the theoretical domain framework [36]. Information is collated on communication styles [26, 27, 33], compliance [30–32, 35, 36], trust and belief systems [28, 29], and interventions to curb pandemics and epidemics [34]. The quality of the studies was assessed in four reviews [29, 31, 34, 35] and three reported risk bias [29, 30, 34].

**Fig. 2:**
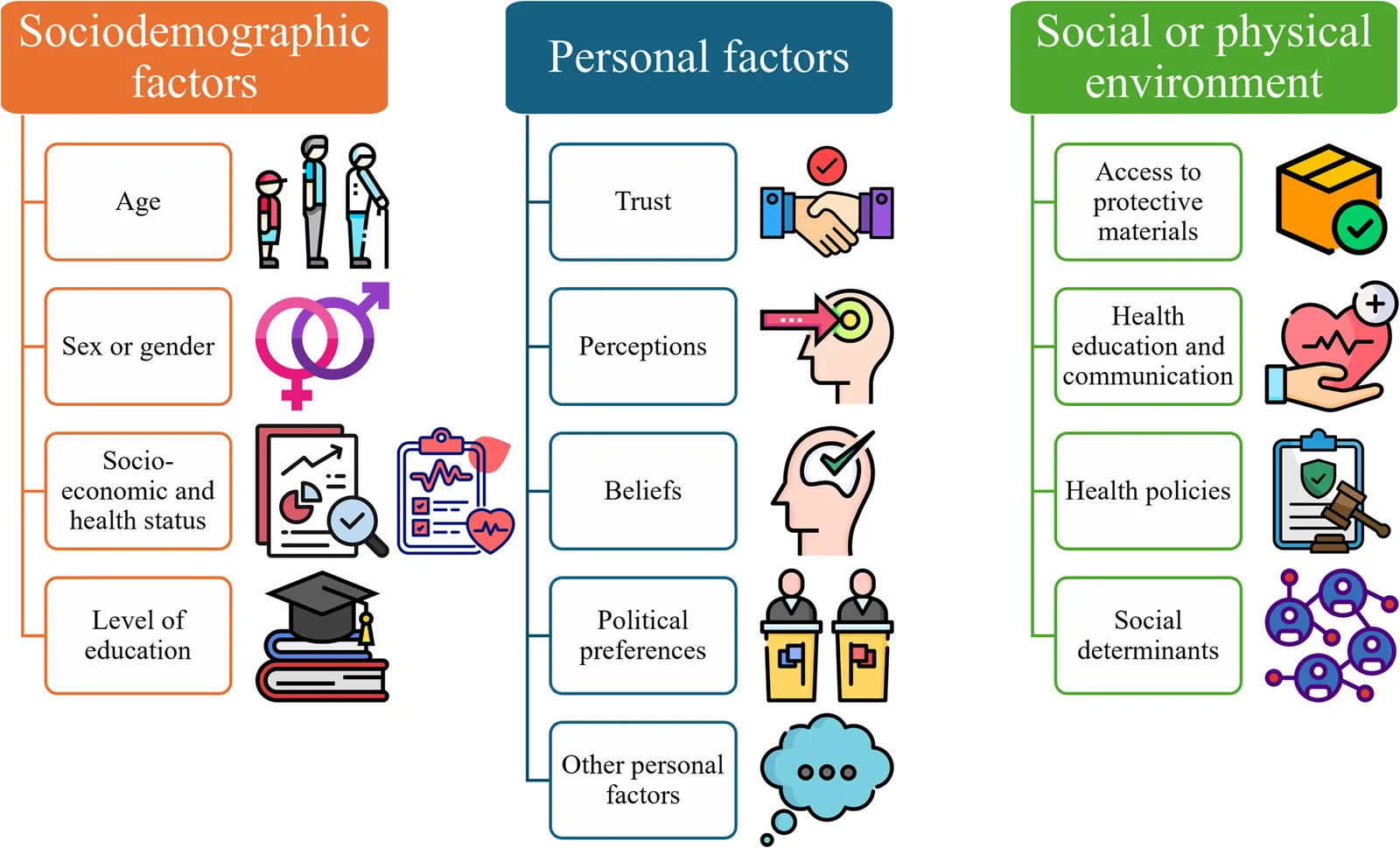
Classification of the factors related to protective behaviours. Icons were retrieved from the Flaticon database accessible at https://www.flaticon.com

Table 2 presents the enabling and hindering factors influencing protective behaviour as well as factors with no relation, categorized into the seven groups of behaviour mentioned above. The subsequent sections elaborate on the influence of the factors regrouped into three categories (Fig. 2) on the relevant protective behaviours.

**Table 2:** Protective behaviours and enabling, hindering or not related factors

Protective Behaviours	Enabling Factors	Hindering Factors
**General protective behaviours/Adherence/Compliance with Public Health recommendations**	Larger households [5] Positive attitude towards isolation [7] Trust in science or medicine [5], in healthcare staff and agencies, in the vaccine + in the majees, in the camp, in Rohingya volunteers [7] WHO recommendation to target refugees for vaccination strategies [7] Recommendations from authorities [11] Perceived effectiveness of guidelines [5] Ability to follow guidelines [5] Social media utilization by health communicators [2] Multimodal communication [1] Access to traditional media sources [5] and to quality information [11], availability of information [7] Working with community/religious leaders [7] Legislation/strict enforcement [7] Acceptance of government restrictions [7] Financial support [11] Social support [in isolation 7; 11] Access to resources [7] Providing information/education, physical opportunity [7] Knowledge about transmission risks [11] Sense of collective responsibility [11] Contact tracing and new cases identification [11] Poor sanitation conditions, access to water, housing [11] Confidence attitude [11] Perception of vulnerability [11]	Political conservatism [5] Negative attitude towards isolation [7] Lack of trust in healthcare staff and agencies, in the vaccine + in the majees, in the camp [7] Conspiracy beliefs [5, 9] COVID-19 is a hoax/caused by 6G > is artificially created or bioweapon [9] Rumours and (religious, personal, or medical) beliefs [7; 11] Watching Fox News [4] Lack of knowledge about diseases [11] Limited availability of information [7] Mosques policies [7] Memory and poor health challenge [7], Forgetfulness [11] Socio-cultural [gender issues] and community norms, unpopularity of certain measures [7] Limited resources (initial lack of vaccine supply), health facilities overwhelmed, limited access (distance and unequal access to health facilities), overcrowded camps, insufficient WASH facilities [7; 11] Risk assessments [7] Shame, fear of stigmatisation and of the pandemic [7; 11] Low perception of susceptibility [11] Lack of social interaction and contact [11] Dissatisfaction with policies, health services and professionals [11] Lack of obligation and monitoring [11] Cultural differences and communication issues [11] Environmental barriers [11]
	*Perceiving COVID-19 as a threat [5/+]* *Influence of family and friends [7]* *Knowledge [7, 5/ +]*	*Influence of family and friends [7]* *Lack of knowledge, of education/literacy skills [7]*
	*Age [older; 5/+]* *Gender [woman or female; 5/+]* *Socio-economic status [high; 5/+]*	*Economic difficulties, financial and food insecurity (i.e., low socio-economic status) [11]*
	*Trust in government [4/+, 5/+]* *Established media outlet communication [10; Dissemination of information through mass media; -]*	
**Medical Interventions**		
Vaccination	Knowledge and experience [3], awareness of the advantages of vaccination [11] Previous vaccination [3], previous successful experience [11] Perception of vulnerability [3] Trust in the vaccine [3, 7] Trust in healthcare staff and agencies + in the majees, in the camp, in Rohingya volunteers [7] Practicability of access and convenience [3; 11] Professional/moral duty [sense of responsibility towards others 3; 11] WHO recommendation to target refugees for vaccination strategies [7] Resolution of the lack of vaccine supply [7] Campaigns, information, media [11] Healthcare professionals guidance [11] Supported or free vaccine [11] Encouragements by health professionals, close people [11]	Poor knowledge [3], misperceptions about vaccines [11] No past vaccination [3], unpleasant previous experience [11] Lack of vulnerability perception [3] Mistrust of/lack of trust in the vaccine [3, 7] Lack of trust in healthcare staff and agencies + in the majees, in the camp [7] Cost [3] Lack of tailored communication mode AND the prioritisation message for ethnic minority groups and migrant people [1] Conspiracy beliefs and their predictors [9] Initial lack of vaccine supply [7] Fake news [11] Limited access [11] Fear of vaccination, injections, pain and side-effects [11] Ethnicity, anti-vaccination group [11] Cultural differences, communication issues [11] Discouragement or lack of recommendation from health professionals, religious leaders, families [11]
Testing	Audio/visual communication [10; safe sex promotion by SMS] Intensive multimedia communication [10; BRIDGE + messages through different mediums] Providing information/education, physical opportunity [7] Working with community/religious leaders [7]	Risk assessment [7] Socio-cultural (gender issues) and community norms, unpopularity of certain measures [7] Shame [7] Limited resources [7]
Treatment	Financial and social support [11] Trust in healthcare staff and agencies, in the vaccine + in the majees, in the camp, in Rohingya volunteers [7] Providing information/education, physical opportunity [7] Practicability of access [11] Recommendation/encouragements by health professionals, close people [11]	Financial and food insecurity Lack of trust in healthcare staff and agencies, in the vaccine + in the majees, in the camp [7] Socio-cultural norms (gender issues) [7] Limited resources [7] Treatment plans [11]
**Conversation promotion**	Intensive multimedia communication [10; education and HIV testing] Peer health communication or education intervention [10; SEPA + cognitive social theory + information + group discussion + role play]	
**Information**		
Knowledge	Trust in formal (governmental) sources [1] Social media utilization by health communicators [2]	
Willingness to provide medical information and receive health services	Trust in health care providers [4]	
Unreliable information and rumours	Mistrust in government [4] Lack of trust in formal (governmental) sources [1] Lack of trust in formal spokespersons [1]	Trust in government [4]
**Hygiene and transmission prevention**		
Hand hygiene/Hygiene	Gender [female or woman; 8] Habits [8] Disgust trigger [8] Social pressure or facilitation [8] Influencers [8] Provision of: hand hygiene products, handwashing exercise, training sessions, posters and stickers; hygiene material, hand hygiene training and information, and diploma; masks and sanitizer with instructions; masks with instructions; sanitizer; sanitizer and hygiene education [6] Health education [6] Hand washing education [6] Request to wash hands, training, poster, instructions [6] Information for children and parents [6] "WHACK the Flu" campaign [6] Web-based sessions [6] Video campaign with social media influencer + article with infographics [5] Moralistic message on duty > virtue [5] Peer health communication or peer health education intervention [10; Online seminars on influenza prevention] Providing information/education, physical opportunity [7] Practicability of access [11]	Environmental constraints [8] Audio/visual communication [10; Addition of health information on posters to prompt hand washing] Unpopularity of certain measures [7]
	*Trust in formal (governmental) sources and authorities [1, 5/+]*	*Provision of masks with use instructions and sanitizer [6/-]* *Conspiracy beliefs [9/+]* *Conspiracy beliefs among other factors [9]*
Use of sanitizer	Provision of: sanitizer; sanitizer, posters, instructions, reminders; sanitizer and use of signs; masks with use instructions and sanitizer; masks and sanitizer with instructions; masks and/or sanitizer and educational intervention [6] "WHACK the Flu" campaign [6]	
Mask	Age [older; 5, 8] Level of education [high; 8] Social pressure or facilitation [8] Awareness of environmental cues [8] Poor self-rated health and/or home duties [8] Perception of susceptibility or severity [8] Hygiene information and mask instructions [6] Health education [6] Legislation/strict enforcement [7] Knowledge about transmission risks [11] Availability [11] Reminders/warnings of the measures [11]	Fear, rumours and (religious or medical) beliefs [7] Community norms, unpopularity of certain measures [7] Limited resources [7] Lack of knowledge about diseases [11] Discomfort and social inconvenience [11]
	*Gender [female or woman; 5/+, 8/+]* *Perception of fatality [8/+]*	*Gender [male or man; 8/+]* *Conspiracy beliefs [9/+]* *Conspiracy beliefs among other factors [9]*
Cough/sneeze cover	Age [older; 8] Gender [female or woman; 8] Level of education [high; 8] Anxiety [8] Perception of susceptibility, severity, or fatality [8] "WHACK the Flu" campaign [6]	
Condom use and safe sex	Intensive multimedia communication [10; Narrative stories "Women's Voices Women's Lives; POWER + phone calls; BRIDGE + messaging through different mediums; MSM targeted ads] Peer health communication or education intervention [10; SEPA + cognitive social theory + information + group discussion + role play; Trained female sex worker education in group]	
	*Audio/visual communication [on condoms discussion, acquisition and use; 10; 3 sessions of education and behavioural modelling/rehearsal, Seminars on HIV/AIDS, SEPA + testing/counselling]* *Peer health communication or education intervention [10/+; trained opinion leader education intervention]*	
**Distancing**		
Distancing/Stay home/Isolation/Quarantine	Trust in healthcare staff and agencies, in the vaccine [7; for refugees: in the majees, in the camp, in Rohingya volunteers] Trust in formal spokespersons [1] Marital status [being married; 8] Concerns about work and finances [8] Family needs [8] Behaviours of others [8] Perception of susceptibility and efficacy [8] Proportionality [8] Legislation/strict enforcement [7] Providing information/education, physical opportunity [7] Reminders/warnings of the measures [11]	Lack of trust in healthcare staff and agencies, in the vaccine [7; for refugees: in the majees, in the camp] Conspiracy beliefs [9] Conspiracy beliefs among other factors [9] Fear, rumours and [religious or medical] beliefs [7] Mosques policies [7] Socio-cultural [gender issues] and community norms, unpopularity of certain measures [7] Limited resources [7] Overcrowded camps [7] Low sense of collective responsibility [11]
	*Level of education [high; 8]* *Trust in the authority [8/-]* *Gender [female; 5/+; 8]*	*Age [young adult; 5/+, 8]*
School closure	Moralistic message on duty or virtue [5]	Belief that it is useless [8] Lack of understanding of the reasons and requirements [8] Perception that one's child is not at risk [8]
Herding	Trust in media [4]	
**Other behaviours**		
Hoarding	Lack of trust in formal (governmental) sources [1] Conspiracy beliefs [9] COVID-19 artificially created > a hoax [9] Conspiracy beliefs and concerns for food shortages [9] Conspiracy beliefs and low income [9] Conspiracy beliefs and dark triad personality traits [9]	
Pseudo-scientific behaviours	Conspiracy beliefs [9]	
Discrimination and prejudice [e.g., against Asian people, food, and restaurants]	Conspiracy beliefs [9]	

Note. The numbers in brackets refer to the reviews from which the information comes: [1] Berg et al., 2021 [26]; [2] Brony et al., 2024 [27]; [3] Hall et al., 2022 [31]; [4] Majid et al., 2021 [28]; [5] Moran et al., 2021 [32]; [6] Perski et al., 2022 [34]; [7] Reda et al., 2025 [35]; [8] Seale et al., 2020 [36]; [9] van Mulukom et al., 2022 [29]; [10] Winograd et al., 2021 [33]; [11] Zaildo et al., 2023 [30]

Factors in italics are controversial (i.e., factors reported in at least two valences). For these, a “+” sign indicates more arguments (i.e., more studies) in favour of the valence of the relation between a factor and a protective behaviour, a “-” sign indicates less arguments (i.e., less studies) and no mention indicates no clear dominance

### Quality of the primary studies and risk of bias assessment

Of the 11 included reviews, only 6 conducted quality appraisal and risk of bias assessment of their included primary studies. Methods used included: JBI checklists [27], Public Health Ontaria Meta QAT tool [31], Cochrane’s risk-of-bias tool [34], the Critical Appraisal Skills Programme [30], the Grading of Recommendations Assessment, Development and Evaluation (GRADE) approach [30, 33], and an assessment based on guidelines [29]. Five reviews did not report conducting formal quality appraisal and risk of bias assessment of primary studies.

Quality ratings of primary studies varied across reviews. Common quality concerns identified across primary studies included sampling bias (convenience sampling or self-selected online samples), measurement issues (inconsistent definitions and measures of protective behaviours), cross-sectional designs, and self-report bias (self-reported rather than observed behaviours).

Regarding the JBI assessment of the reviews (see Supplementary File 4), the criteria for appraising studies were frequently inappropriate or unclear. The critical appraisal was not conducted by two or more reviewers independently, and there was a lack of methods to minimize errors in data extraction. Factors were also stratified by type of review for transparency regarding the rigour of their methodology (see Supplementary File 5).

However, the heterogeneity of appraisal tools, criteria, and reporting practices across reviews made it difficult to compare quality ratings or to synthesise them meaningfully. Given that only about half of the included reviews conducted a formal assessment—and those that did used different frameworks with varying scoring systems and thresholds—no consistent pattern emerged that could be reliably integrated into the overall interpretation of findings. In addition, several reviews provided narrative judgements without quantitative ratings, further limiting comparability.

### Weight of the results

The valence (enabling, hindering or without effect) of the results reported in Table 2 were decided according to the valence reported in each review. Controversial results were mainly resolved by the number of reviews and of studies in favour of an interpretation of the valence (see + and – symbols in Table 2). Consistent, directionally aligned results (e.g., female enabling / male hindering) were treated as coherent patterns and not subject to weighting rules. Eventually, the contradictory results were put into context to justify the discrepancy (e.g., audio/visual communication has a positive effect on condoms discussion, acquisition and use, and has no relation with the number of partners).

The quality of the reviews was satisfactory (mean JBI score 8.8/10; range 7–10). For the reasons mentioned in the previous section, although quality appraisal and risk of bias assessments were considered, they were not used as a basis for weighting evidence in the present synthesis. Instead, the results are interpreted with an overarching awareness of the methodological limitations commonly noted across primary studies (e.g., sampling and measurement issues), while avoiding potentially misleading comparisons arising from incompatible evaluation approaches.

### Sociodemographic factors

#### Age

Although some studies indicate that age is not relevant (i.e., no relation), most of the studies included in the review indicate that older individuals exhibit enhanced compliance [32]. Elderly people seems also to be more prone to adopt protective behaviours such as cough/sneeze cover and mask wearing [32, 36], while younger adults demonstrate lower compliance with distancing directives [32, 36], even if few data does not indicate any impact [32]. Results about age-related impacts on hygiene tends to show no significant relation.

#### Sex or gender

Female individuals are linked to higher compliance and adoption of protective behaviours such as hygiene, transmission prevention, and distancing measures [32, 36], whereas males seems to be less likely to wear masks [36]. In a lesser extent (i.e., less documented) some findings suggest no effect of sex or gender on compliance and protective behaviour adoption, specifically regarding mask use and distancing [33, 36].

#### Socio-economic and health status

Higher socio-economic status, contrary to financial and food insecurity, and larger household size, correlate with greater compliance and adoption of protective behaviours [30, 32]. Factors such as marital status and financial concerns tends to facilitate compliance with distancing directives [36]; however, some studies indicate, in a lesser extent, no relation between socio-economic status and compliance [32]. Furthermore, employment and health status do not seem to influence how well people follow protective behaviours overall [32]. Similarly, employment and household status are not related to hygiene practices, and socio-economic status show no significant impact on mask usage [32]. Additionally, race, ethnicity, household structure, socio-economic status, employment, or health status are not associated with compliance with distancing guidelines [32].

#### Level of education

Educational attainment did not show influence on overall compliance or hygiene practices [32, 34] but is positively associated with mask-wearing and respiratory etiquette [36]. One broader review—including SARS, MERS, H1N1, COVID-19, and hypothetical pandemics—suggested that higher education fosters compliance with distancing rules [36], while another focussing on COVID-19 deemed it non-influential on such behaviours [32]. Knowledge and experience tends to foster vaccination, while inadequate information or fake news seems to hinder it in general population [30], among healthcare workers [31], and among minority and migrant populations [26]. Knowledge is related to improvement of general compliance [30, 35] although a few lesser studies do not find any effect [32]. Awareness of public health guidelines does not necessarily translate to compliance with protective behaviours [32], and vaccine education alone does not guarantee uptake among healthcare workers [31].

### Personal factors

#### Trust

Trust can pertain to various entities, including authorities, science, medicine, healthcare staff and agencies, and media. Trust in *authorities* and *healthcare staff* correlates with general compliance [28, 32, 35], despite some minority studies indicating no relation [28, 32]. Trust may mitigate misinformation, enable knowledge [26, 28] and foster compliance with directives. A deficit in trust is linked to hoarding behaviours [26]. Trust in *science and medicine* tends to encourage compliance and willingness to engage with health services [26, 28] but does not influence compliance with distancing measures [32]. Trust in COVID-19 *vaccines* seems to enhance vaccination rates, while mistrust is related to a decrease in general population [30] and among healthcare workers [31]. Trust in *media* may encourage herding behaviour. Conversely, trust in *others* does not seem to affect compliance or protective behaviour adoption [32].

#### Perceptions

Perceived effectiveness of guidelines tends to enhance compliance [32]. The perception of COVID-19 as a threat rather seems to enable compliance despite few studies asserting no significant impact on [32]. Perceived threat levels of COVID-19 do not show any effect on hygiene practices or compliance with distancing directives [32].

Perceptions of susceptibility and severity are related to compliance with general protective behaviours [30], distancing, and hygiene practices [36], although lack of knowledge about fatality rates does not seem impact mask-wearing [36]. Vulnerability perceptions and professional duty tend to enhance vaccination willingness among healthcare workers [31]. Acceptance of school closures is challenging for those who view them as ineffective and think their children are at low risk [36].

#### Beliefs

Conspiracy beliefs is related to the reduction of various protective behaviours, including compliance [29, 32], despite some studies finding no relation [32]. These beliefs tend to negatively impact vaccination rates, mask-wearing, and hygiene practices, although some studies report no relation. Furthermore, conspiracy beliefs may promote alternative protective actions such as hoarding, discrimination, and pseudo-scientific practices involving alternative remedies [29]. Religious, personal or medical beliefs also reduce general adherence to recommendations [30, 35] and to mask wearing among refugees [35].

#### Political preferences

Political conservatism seems to impede general compliance but politics do not show influence on mask wearing or respect for distancing directives [32].

#### Other personal factors

Habits and past behaviours (previous vaccination) are related to the increase of vaccination among healthcare workers [31] and hand and general hygiene [36]. Compliance with guidelines [32] and positive attitude towards isolation [35] correlates with the adoption of protective behaviours. Anxiety is linked to cough or sneeze cover, and disgust generates hand and general hygiene [36].

### Social or physical environment

#### Access to protective materials

Provision of and access to resources, hygiene materials, sanitizers and masks, seems to be of importance for general adherence to recommendations, hand and general hygiene, and sanitizer use in general population [34], among healthcare workers [31], and among refugees [35]; however, one primary study of a review [34] indicated mixed results regarding the provision of mask with use instructions and sanitizer on hygiene practices.

#### Health education and communication

Educational interventions (e.g., hygiene education, media campaigns, training sessions, posters, etc.) are generally related to more testing [35], hand and general hygiene [32, 34], mask-wearing, use of sanitizers, cough and sneeze cover [34] testing, and open dialogue promotion [33]. However, few interventions report no or negative effects [33, 34].

Multimodal communication and access to traditional media are linked to enhanced compliance [26, 27, 30, 32, 35], while moralistic messaging tends to enable acceptance of school closures but does not impact compliance with distancing [32].

#### Health policies

Recommendations from authorities and legislation/obligation with monitoring seems to promote general compliance [30] and vaccine uptake among refugees [35]. Financial support is related to adherence, vaccination, and treatment in general population [30] and among healthcare workers [31].

#### Social determinants

Social pressure or influencers, and peer communication may affect general compliance in both ways among refugees (depending on the opinion of influencers and peers; 35) but seems to improve compliance with hygiene practices, open dialogue promotion, and quarantine compliance [33, 36]. Socio-cultural and community norms is negatively associated with global adherence, distancing measures, and testing propensity among refugees [35], while ethnicity and anti-vaccination groups reduced vaccination rate [30]. The sense of collective responsibility is identified as a enabling factor of general compliance, vaccination, and distancing among healthcare workers [31] and refugees [35]. Social support tends to improve adherence to measures and treatment [30].

## Discussion

Our umbrella review aimed to address several gaps from the existing literature despite the proliferation of review on COVID-19. We synthesized evidence across behaviours and contexts, identified converging and diverging findings, assessed the quality of the evidence, and provided guidance for pandemic preparedness.

### Main findings of this study

This investigation identified key strategies to promote protective behaviours during pandemics. These include targeting non-compliant populations, enhancing trust in authorities and scientific evidence, addressing conspiracy theories, disseminating tailored information and educational resources, and ensuring access to hygiene materials. This study synthesized findings from eleven reviews that examined seven distinct protective behaviours: overall compliance with protective measures, medical interventions (including vaccination, testing, and treatment), open dialogue promotion, information acquisition, (hand) hygiene, physical distancing, and “others”. These behaviours are influenced by three categories of factors. *Socio-demographic factors* (i.e., age, sex or gender, socio-economic and health status, and level of education) are not directly amendable by policies but must be acknowledged to identify hesitant populations for intervention. *Personnal factors* (i.e., trust, perceptions, beliefs, political inclinations, and other personal elements) constitute the individual variables that could be targeted through communication campaigns and policies. The *social or physical environment* (i.e., accessibility to protective materials, health education and communication, health policies, and social determinants) encompasses overarching contextual elements that can also be enhanced for improved compliance.

The strategies proposed in this review directly target the synthesized determinants. Specifically: (1) targeting non-compliant populations addresses sociodemographic variations in adherence; (2) enhancing trust targets the psychological factor of trust in authorities and science; (3) addressing conspiracy theories targets the psychological barrier of misinformation beliefs; (4) disseminating tailored information targets knowledge gaps and risk perceptions; and (5) ensuring access to hygiene materials addresses the environmental barrier of resource accessibility. These strategy-determinant links are grounded in the consistent findings across included reviews showing that trust, accurate information, and resource access are key enablers of protective behaviours [28, 30, 32, 34].

The reviews emphasized the importance of transparent, timely communication and health education. Effective messaging should integrate risk alerts with actionable advice, clarify evolving recommendations, and offer alternatives to risky behaviours. Rapid dissemination of new information and correction of earlier messages are also crucial [32, 37, 38].

To clarify our conceptual framework: the seven protective behaviours identified (compliance, medical interventions, open dialogue promotion, information acquisition, hygiene, physical distancing, and others) represent observable behavioural domains reported across included reviews. These are distinct from their determinants (the factors that enable or hinder them, such as trust and risk perception), interventions (deliberate programs designed to change behaviour, such as education campaigns), and maladaptive responses (counterproductive reactions such as conspiracy beliefs that reduce compliance). This classification reflects how behaviours were categorized in the original reviews and allows for behaviour-specific analysis of determinants.

### What is already known on this topic

The COVID-19 pandemic highlighted the vital role of protective behaviours—such as mask-wearing, hand hygiene, physical distancing, and isolation—in limiting disease transmission [39]. Evidence from previous pandemics (H1N1, SARS, MERS, Ebola) has consistently demonstrated that successful adoption of protective behaviours depends on multiple interconnected factors spanning individual, social, and structural levels. Theoretical frameworks including the Health Belief Model [40], Theory of Planned Behaviour [41], and Capability-Opportunity-Motivation-Behaviour model [42] have identified risk perception, self-efficacy, social norms, and outcome expectations as key determinants. Our synthesis maps onto these frameworks: personnal factors (trust, perceptions, beliefs) correspond to Health Belief Model and Theory of Planned Behaviour constructs of perceived threat and attitudes; social environment factors (social norms, community support) correspond to Theory of Planned Behaviour’s subjective norms and Capability-Opportunity-Motivation-Behaviour’s social opportunity; and access to protective materials corresponds to Capability-Opportunity-Motivation-Behaviour’s physical opportunity. By synthesizing across multiple theoretical models, this review identifies convergent determinants (e.g., self-efficacy and trust appear across all frameworks) and highlights environmental factors often underemphasized in individually-focused models like Health Belief Model and Theory of Planned Behaviour.

Prior research has also shown that beyond knowledge, factors like social cohesion, trust in public authorities, and perceived government competence are essential for effective crisis response. Communication strategies, information source credibility, and message framing significantly influence behaviour adoption across diverse populations. However, existing reviews have typically focused on single behaviours or specific populations, and studies yield inconsistent results due to methodological variations and cultural differences. A comprehensive understanding of the enabling and inhibiting factors across different behavioural domains remained fragmented, limiting the development of unified, evidence-based frameworks for pandemic preparedness and targeted policy guidance across diverse health crisis contexts.

### What this study adds

#### Evidence-supported findings

This umbrella review consolidates insights from eleven reviews into a unified framework, synthesizing protective and obstructive factors across seven behavioural domains. The synthesis provides three evidence-based conclusions. First, protective behaviours during pandemics are influenced by multi-level determinants spanning sociodemographic characteristics, personnal factors (trust, perceptions, beliefs), and environmental factors (resource access, social norms, communication strategies). This multi-level pattern was consistent across all included reviews [26, 36]. Second, certain determinants showed consistent effects across multiple behaviours and studies: trust in authorities and scientific evidence, perceived effectiveness of protective measures, access to accurate information, and availability of protective resources were consistently associated with higher adherence [28, 30, 32, 34]. Conversely, conspiracy beliefs, misinformation, and resource inaccessibility consistently hindered protective behaviours [29, 30]. Third, some determinants (age, gender, education, socioeconomic status) showed inconsistent effects across behaviours and contexts, suggesting that their influence is behaviour-specific or moderated by cultural and contextual factors [26, 32, 34].

#### Interpretive implications and recommendations

Based on these patterns, we propose the following implications for policy and practice, recognizing that these represent interpretive extrapolations requiring further testing:

The multi-level framework enables targeted resource allocation and intervention design. Common facilitators across behaviours (trust, risk perception, social norms, self-efficacy) suggest that integrated policy approaches may be more cost-effective than behaviour-specific interventions. Demographic and socioeconomic variations highlight critical health equity considerations, demanding culturally sensitive and inclusive strategies. Effective communication requires transparent, timely, and scientifically accurate messaging through trusted channels. Future pandemic preparedness should combine communication, regulation, incentives, and social influence strategies while balancing individual autonomy with collective responsibility.

### Limitations of this study

This umbrella review has several limitations that should be considered when interpreting findings.

We excluded grey literature and studies on pandemics other than COVID-19, although some of the selected reviews included grey literature and other infectious diseases. Our focused search strategy (“COVID-19” AND “protective behaviours”) may have missed reviews using alternative terminology (e.g., preventive behaviours, adherence, distancing), though our search yielded significant results answering the question of this paper. This trade-off between feasibility and comprehensiveness is related to umbrella review methodology but may affect completeness of our synthesis.

This review is limited to peer-reviewed literature in English, French, and Dutch and included reviews predominantly synthesized evidence from high-income, Western countries (North America, Europe, Australia). Evidence from low- and middle-income countries, particularly in Africa, South America, and parts of Asia, was limited. This geographic bias limits generalizability of findings to non-Western contexts where determinants may differ due to cultural, economic, and health system factors.

We were unable to fully quantify the degree of overlap in primary studies across included reviews due to incomplete citation data in some reviews. Based on manual inspection of available citations, we observed apparent moderate overlap, particularly among reviews focusing on COVID-19 protective behaviours. This means some primary studies may have contributed to multiple reviews in our synthesis, potentially amplifying certain findings. We attempted to account for this by prioritizing the most recent and comprehensive reviews when findings converged.

Several of the reviews [28, 29, 31, 33] did not assess publication bias in their primary studies. They also pointed out the poor quality of studies they included and noted the lack of comparative, experimental, or longitudinal designs. Although they were put into perspective in relation to the number of primary studies, inconsistencies must also be acknowledged (see factors in italics in Table 2; they may be due to different definitions of the factors across included studies and methodological limitations (e.g., cross-sectional designs, convenience sampling, lack of sound statistical analyses, adoption of single vs. multiple behaviours). The discrepancies may indicate that factors are interconnected and that they should be considered as systems, potentially with network analyses, instead of separately. These gaps highlight the need for more robust research.

Observed heterogeneity may be explained by differences in sociodemographic profiles, trust levels, cultural norms, and resource availability. Methodological diversity (e.g., differences in the definition and the assessment of “protective behaviours” and pandemic context further contribute to variability. Moreover, due to the design of primary studies, causality cannot be established, requiring further longitudinal and experimental evidence.

Additionally, while this review offers strategic insights, it cannot fully account for the evolution of the factors’ influences across pandemic phases (early outbreak, peak transmission, endemic transition) and the context-specific nature of public health crises. Future research should explore what factors are more important for each phase, and mobilize social capital to enhance societal resilience.

Despite these limitations, the findings can inform emergency preparedness plans, including simulations, resource distribution, and coordination with local authorities [14, 15, 34, 36], and support prevention strategies that empower individuals and communities to navigate uncertainty [5–8].

## Supplementary Information

Supplementary Material 1.

Supplementary Material 2.

Supplementary Material 3.

Supplementary Material 4.

Supplementary Material 5.

Supplementary Material 6.

## Data Availability

Any data that could not be found in the supplementary files can be obtained upon reasonable request to the corresponding author.
