# Supplementary material for: Key factors for promoting protective behaviours in future pandemics: an umbrella review
Source: Arch Public Health. 2026 Jun 17;84:200. doi: 10.1186/s13690-026-01948-6 (PMC13573413; doi:10.1186/s13690-026-01948-6)
Supplement: Supplementary file 2 [file 13690_2026_1948_MOESM2_ESM.docx]

Search Strategy

- Search run 30/11/2023

*Database 1: Pubmed*

Query: "COVID-19"[Title/Abstract] AND "PROTECTIVE BEHAVIO*"[Title/Abstract] AND (meta-analysis[Filter] OR review[Filter] OR systematicreview[Filter])

Results: 24

*Database 2: Scopus*

Query: (TITLE-ABS-KEY(covid-19) AND TITLE-ABS-KEY("protective behavio*")) AND ( LIMIT-TO ( DOCTYPE,"re" ) )

Results: 24

Duplicates: 15

- Search update 31/03/2024

Pubmed Results: 4

Scopus Results: 3

Duplicates: 2

- Search update 30/06/2025

Pubmed Results: 4

Scopus Results: 4

*Database 3: Epistemonikos*

Query: “(title:COVID-19 OR abstract:COVID-19) AND ((title:"protective behavior" OR abstract:"protective behavior") OR (title:"protective behaviour" OR abstract:"protective behaviour") OR (title:"protective behaviors" OR abstract:"protective behaviors") OR (title:"protective behaviours" OR abstract:"protective behaviours"))”

Filter: Publication type: “Systematic Review”

Results: 23

Duplicates: 16
