# Supplementary material for: Key factors for promoting protective behaviours in future pandemics: an umbrella review
Source: Arch Public Health. 2026 Jun 17;84:200. doi: 10.1186/s13690-026-01948-6 (PMC13573413; doi:10.1186/s13690-026-01948-6)
Supplement: Supplementary file 3 [file 13690_2026_1948_MOESM6_ESM.docx]

**Table 2** Protective behaviours and enabling, hindering or not related factors.

| Protective Behaviours | Enabling Factors | Hindering Factors | Factors reported as having no relation |
| --- | --- | --- | --- |
| **General protective behaviours/Adherence/Compliance with Public Health recommendations** | Larger households [5] Positive attitude towards isolation [7] Trust in science or medicine [5], in healthcare staff and agencies, in the vaccine + in the majees, in the camp, in Rohingya volunteers [7] WHO recommendation to target refugees for vaccination strategies [7] Recommendations from authorities [11] Perceived effectiveness of guidelines [5] Ability to follow guidelines [5] Social media utilization by health communicators [2]  Multimodal communication [1] Access to traditional media sources [5] and to quality information [11], availability of information [7] Working with community/religious leaders [7] Legislation/strict enforcement [7] Acceptance of government restrictions [7] Financial support [11] Social support [in isolation 7; 11]] Access to resources [7] Providing information/education, physical opportunity [7] Knowledge about transmission risks [11] Sense of collective responsibility [11] Contact tracing and new cases identification [11] Poor sanitation conditions, access to water, housing [11] Confidence attitude [11] Perception of vulnerability [11] | Political conservatism [5] Negative attitude towards isolation [7] Lack of trust in healthcare staff and agencies, in the vaccine + in the majees, in the camp [7] Conspiracy beliefs [5, 9]  COVID-19 is a hoax/caused by 6G > is  artificially created or bioweapon [9] Rumours and (religious, personal, or medical) beliefs [7; 11] Watching Fox News [4] Lack of knowledge about diseases [11] Limited availability of information [7] Mosques policies [7] Memory and poor health challenge [7], Forgetfulness [11] Socio-cultural [gender issues] and community norms, unpopularity of certain measures [7] Limited resources (initial lack of vaccine supply), health facilities overwhelmed, limited access (distance and unequal access to health facilities), overcrowded camps, insufficient WASH facilities [7; 11] Risk assessments [7] Shame, fear of stigmatisation and of the pandemic [7; 11] Low perception of susceptibility [11] Lack of social interaction and contact [11] Dissatisfaction with policies, health services and professionals [11] Lack of obligation and monitoring [11] Cultural differences and communication issues [11] Environmental barriers [11] | Employment, and health status [5]  Beliefs in conspiracy theories [5]  Trust in others [5]  Education [5] |

*(Continued)*

**Table 2** Continued.

| Protective Behaviours | Enabling Factors | Hindering Factors | Factors reported as having no relation |
| --- | --- | --- | --- |
| **General protective behaviours/Adherence/Compliance with Public Health recommendations [continued]** | *Perceiving COVID-19 as a threat [5/+] Influence of family and friends [7] Knowledge [7, 5/ +]  Age [older; 5/+] Gender [woman or female; 5/+] Socio-economic status [high; 5/+]   Trust in government [4/+, 5/+] Established media outlet communication [10; Dissemination of information through mass media; -]* | *Influence of family and friends [7] Lack of knowledge, of education/literacy skills [7]   Economic difficulties, financial and food insecurity (i.e., low socio-economic status) [11]* | *Perceiving COVID-19 as a threat [5/-]  Knowledge about pandemic or public health guidelines [5/-] Age [5/-] Sex/Gender [5/-] Socio-economic status [5/-]   Trust in government or authorities [4/-, 5/-] Established media outlet communication [10; Dissemination of information through mass media; +]* |
| **Medical Interventions** |  |  |  |
| Vaccination | Knowledge and experience [3], awareness of the advantages of vaccination [11] Previous vaccination [3], previous successful experience [11] Perception of vulnerability [3] Trust in the vaccine [3, 7] Trust in healthcare staff and agencies + in the majees, in the camp, in Rohingya volunteers [7] Practicability of access and convenience [3; 11] Professional/moral duty [sense of responsibility towards others 3; 11] WHO recommendation to target refugees for vaccination strategies [7] Resolution of the lack of vaccine supply [7] Campaigns, information, media [11] Healthcare professionals guidance [11] Supported or free vaccine [11] Encouragements by health professionals, close people [11] | Poor knowledge [3], misperceptions about vaccines [11]  No past vaccination [3], unpleasant previous experience [11] Lack of vulnerability perception [3] Mistrust of/lack of trust in the vaccine [3, 7] Lack of trust in healthcare staff and agencies + in the majees, in the camp [7] Cost [3] Lack of tailored communication mode AND the prioritisation message for ethnic minority groups and migrant people [1] Conspiracy beliefs and their predictors [9] Initial lack of vaccine supply [7] Fake news [11] Limited access [11] Fear of vaccination, injections, pain and side-effects [11] Ethnicity, anti-vaccination group [11] Cultural differences, communication issues [11] Discouragement or lack of recommendation from health professionals, religious leaders, families [11] | Vaccination education alone [3] |

*(Continued)*

**Table 2** Continued.

| Protective Behaviours | Enabling Factors | Hindering Factors | Factors reported as having no relation |
| --- | --- | --- | --- |
| **Medical Interventions [continued]** |  |  |  |
| Testing | Audio/visual communication [10; safe sex promotion by SMS] Intensive multimedia communication [10; BRIDGE + messages through different mediums] Providing information/education, physical opportunity [7] Working with community/religious leaders [7] | Risk assessment [7] Socio-cultural (gender issues) and community norms, unpopularity of certain measures [7] Shame [7] Limited resources [7] |  |
| Treatment | Financial and social support [11] Trust in healthcare staff and agencies, in the vaccine + in the majees, in the camp, in Rohingya volunteers [7] Providing information/education, physical opportunity [7] Practicability of access [11] Recommendation/encouragements by health professionals, close people [11] | Financial and food insecurity Lack of trust in healthcare staff and agencies, in the vaccine + in the majees, in the camp [7] Socio-cultural norms (gender issues) [7] Limited resources [7] Treatment plans [11] |  |
| **Conversation promotion** | Intensive multimedia communication [10; education and HIV testing]  Peer health communication or education intervention [10; SEPA + cognitive social theory + information + group discussion + role play] |  |  |
| **Information** |  |  |  |
| Knowledge | Trust in formal (governmental) sources [1] Social media utilization by health communicators [2] |  |  |
| Willingness to provide medical information and receive health services | Trust in health care providers [4] |  |  |
| Unreliable information and rumours | Mistrust in government [4] Lack of trust in formal (governmental) sources [1] Lack of trust in formal spokespersons [1] | Trust in government [4] |  |

*(Continued)*

**Table 2** Continued.

| Protective Behaviours | Enabling Factors | Hindering Factors | Factors reported as having no relation |
| --- | --- | --- | --- |
| **Hygiene and transmission prevention** |  |  |  |
| Hand hygiene/Hygiene | Gender [female or woman; 8] Habits [8] Disgust trigger [8] Social pressure or facilitation [8] Influencers [8] Provision of: hand hygiene products, handwashing exercise, training sessions, posters and stickers; hygiene material, hand hygiene training and information, and diploma; masks and sanitizer with instructions; masks with instructions; sanitizer; sanitizer and hygiene education [6] Health education [6] Hand washing education [6] Request to wash hands, training, poster, instructions [6] Information for children and parents [6] "WHACK the Flu" campaign [6] Web-based sessions [6] Video campaign with social media influencer + article with infographics [5] Moralistic message on duty > virtue [5] Peer health communication or peer health education intervention [10; Online seminars on influenza prevention] Providing information/education, physical opportunity [7] Practicability of access [11] | Environmental constraints [8] Audio/visual communication [10; Addition of health information on posters to prompt hand washing] Unpopularity of certain measures [7] | Age [5]  Education [5,6]  Employment status [5]  Household status [5]  Perceiving COVID-19 as a threat [5]  Influenza Prevention Prescription  Information about hands cleaning and planning task [6]  Audio/visual communication [10; Influenza messaging during controlled health record] |
|  | *Trust in formal (governmental) sources and authorities [1, 5/+]* | *Provision of masks with use instructions and sanitizer [6/-] Conspiracy beliefs [9/+]  Conspiracy beliefs among other factors [9]* | *Trust in government or authorities, and others [5/-] Provision of masks with use instructions and sanitizer [6/+]*  *Conspiracy beliefs [9/-]* |

*(Continued)*

**Table 2** Continued.

| Protective Behaviours | Enabling Factors | Hindering Factors | Factors reported as having no relation |
| --- | --- | --- | --- |
| **Hygiene and transmission prevention [continued]** |  |  |  |
| Use of sanitizer | Provision of: sanitizer; sanitizer, posters, instructions, reminders; sanitizer and use of signs; masks with use instructions and sanitizer; masks and sanitizer with instructions; masks and/or sanitizer and educational intervention [6]   "WHACK the Flu" campaign [6] |  |  |
| Mask | Age [older; 5, 8] Level of education [high; 8] Social pressure or facilitation [8] Awareness of environmental cues [8] Poor self-rated health and/or home duties [8] Perception of susceptibility or severity [8] Hygiene information and mask instructions [6] Health education [6] Legislation/strict enforcement [7] Knowledge about transmission risks [11] Availability [11] Reminders/warnings of the measures [11] | Fear, rumours and (religious or medical) beliefs [7] Community norms, unpopularity of certain measures [7] Limited resources [7] Lack of knowledge about diseases [11] Discomfort and social inconvenience [11] | Socio-economic status [5] Politics [5] |
|  | *Gender [female or woman; 5/+, 8/+] Perception of fatality [8/+]* | *Gender [male or man; 8/+]  Conspiracy beliefs [9/+] Conspiracy beliefs among other factors [9]* | *Sex/Gender [5/-, 8/-] Lack of knowledge about fatality rate [8/-] Conspiracy beliefs [9/-]* |
| Cough/sneeze cover | Age [older; 8] Gender [female or woman; 8] Level of education [high; 8] Anxiety [8] Perception of susceptibility, severity, or fatality [8] "WHACK the Flu" campaign [6] |  | Audio/visual communication [10; Influenza messaging during controlled health record] |

*(Continued)*

**Table 2** Continued.

| Protective Behaviours | Enabling Factors | Hindering Factors | Factors reported as having no relation |
| --- | --- | --- | --- |
| **Hygiene and transmission prevention [continued]** |  |  |  |
| Condom use and safe sex | Intensive multimedia communication [10; Narrative stories "Women's Voices Women's Lives; POWER + phone calls; BRIDGE + messaging through different mediums; MSM targeted ads] Peer health communication or education intervention [10; SEPA + cognitive social theory + information + group discussion + role play; Trained female sex worker education in group] |  | Intensive multimedia communication [10; education and HIV testing] |
|  | *Audio/visual communication [on condoms discussion, acquisition and use; 10; 3 sessions of education and behavioural modelling/rehearsal, Seminars on HIV/AIDS, SEPA + testing/counselling]*  *Peer health communication or education intervention [10/+; trained opinion leader education intervention]* |  | *Audio/visual communication [on number of partners; 10; 3 sessions of education and behavioural modelling/rehearsal, Seminars on HIV/AIDS, SEPA + testing/counselling]  Peer health communication or education intervention [10/-; trained opinion leader education intervention]* |
| **Distancing** |  |  |  |
| Distancing/Stay home/Isolation/Quarantine | Trust in healthcare staff and agencies, in the vaccine [7; for refugees: in the majees, in the camp, in Rohingya volunteers] Trust in formal spokespersons [1] Marital status [being married; 8] Concerns about work and finances [8] Family needs [8] Behaviours of others [8] Perception of susceptibility and efficacy [8] Proportionality [8] Legislation/strict enforcement [7] Providing information/education, physical opportunity [7] Reminders/warnings of the measures [11] | Lack of trust in healthcare staff and agencies, in the vaccine [7; for refugees: in the majees, in the camp] Conspiracy beliefs [9] Conspiracy beliefs among other factors [9]  Fear, rumours and [religious or medical] beliefs [7] Mosques policies [7] Socio-cultural [gender issues] and community norms, unpopularity of certain measures [7] Limited resources [7] Overcrowded camps [7] Low sense of collective responsibility [11] | Trust in science, and in others [5] Race or ethnicity [5] Socio-economic, employment, and health status [5] Household structure [5] Perceiving COVID-19 as a threat, and protective behaviours as effective [5] Politics [5] Moralistic message on duty or virtue [5] |
|  | *Level of education [high; 8] Trust in the authority [8/-] Gender [female; 5/+; 8]* | *Age [young adult; 5/+, 8]* | *Education [5] Trust in government or authorities [5/+] Sex/Gender [5/-] Age [5/-]* |

*(Continued)*

**Table 2** Continued.

| Protective Behaviours | Enabling Factors | Hindering Factors | Factors reported as having no relation |
| --- | --- | --- | --- |
| **Distancing [continued]** |  |  |  |
| School closure | Moralistic message on duty or virtue [5] | Belief that it is useless [8] Lack of understanding of the reasons and requirements [8] Perception that one's child is not at risk [8] |  |
| Herding | Trust in media [4] |  |  |
| **Other behaviours** |  |  |  |
| Hoarding | Lack of trust in formal (governmental) sources [1] Conspiracy beliefs [9]  COVID-19 artificially created > a hoax [9] Conspiracy beliefs and concerns for food shortages [9] Conspiracy beliefs and low income [9] Conspiracy beliefs and dark triad personality traits [9] |  |  |
| Pseudo-scientific behaviours | Conspiracy beliefs [9] |  |  |
| Discrimination and prejudice [e.g., against Asian people, food, and restaurants] | Conspiracy beliefs [9] |  |  |

*Note. The numbers in brackets refer to the reviews from which the information comes: [1] Berg et al., 2021 (26); [2] Brony et al., 2024 (27); [3] Hall et al., 2022 (31); [4] Majid et al., 2021 (28); [5] Moran et al., 2021 (32); [6] Perski et al., 2022 (34); [7] Reda et al., 2025 (29); [8] Seale et al., 2020 (36); [9] van Mulukom et al., 2022 (29); [10] Winograd et al., 2021 (33); [11] Zaildo et al., 2023 (30). Factors in italics are controversial (i.e., factors reported in at least two valences). For these, a “+” sign indicates more arguments (i.e., more studies) in favour of the valence of the relation between a factor and a protective behaviour, a “-” sign indicates less arguments (i.e., less studies) and no mention indicates no clear dominance.*
